# Hydrophobic Coatings by Thiol-Ene Click Functionalization of Silsesquioxanes with Tunable Architecture

**DOI:** 10.3390/ma10080913

**Published:** 2017-08-08

**Authors:** Sandra Dirè, Davide Bottone, Emanuela Callone, Devid Maniglio, Isabelle Génois, François Ribot

**Affiliations:** 1 Department of Industrial Engineering, University of Trento, via Sommarive 9, 30123 Trento, Italy; emanuela.callone@unitn.it (E.C.); devid.maniglio@unitn.it (D.M.); 2 Sorbonne Universités, UPMC University Paris 06—CNRS—College de France, UMR 7574, Laboratoire de Chimie de la Matière Condensée de Paris, 4 place Jussieu, 75005 Paris, France; isabelle.genois@upmc.fr (I.G.); francois.ribot@upmc.fr (F.R.)

**Keywords:** silsesquioxanes, thiol-ene click reaction, in situ water production, hydrophobic coatings, cotton fabric, paper, NMR, wettability

## Abstract

The hydrolysis-condensation of trialkoxysilanes under strictly controlled conditions allows the production of silsesquioxanes (SSQs) with tunable size and architecture ranging from ladder to cage-like structures. These nano-objects can serve as building blocks for the preparation of hybrid organic/inorganic materials with selected properties. The SSQs growth can be tuned by simply controlling the reaction duration in the in situ water production route (ISWP), where the kinetics of the esterification reaction between carboxylic acids and alcohols rules out the extent of organosilane hydrolysis-condensation. Tunable SSQs with thiol functionalities (SH-NBBs) are suitable for further modification by exploiting the simple thiol-ene click reaction, thus allowing for modifying the wettability properties of derived coatings. In this paper, coatings were prepared from SH-NBBs with different architecture onto cotton fabrics and paper, and further functionalized with long alkyl chains by means of initiator-free UV-induced thiol-ene coupling with 1-decene (C10) and 1-tetradecene (C14). The coatings appeared to homogeneously cover the natural fibers and imparted a multi-scale roughness that was not affected by the click functionalization step. The two-step functionalization of cotton and paper warrants a stable highly hydrophobic character to the surface of natural materials that, in perspective, suggests a possible application in filtration devices for oil-water separation. Furthermore, the purification of SH-NBBs from ISWP by-products was possible during the coating process, and this step allowed for the fast, initiator-free, click-coupling of purified NBBs with C10 and C14 in solution with a nearly quantitative yield. Therefore, this approach is an alternative route to get sol-gel-derived, ladder-like, and cage-like SSQs functionalized with long alkyl chains.

## 1. Introduction

Hybrid organic-inorganic (O/I) materials combine the characteristics of their organic and inorganic components to obtain mechanical, electrical, and chemical properties that would not be achievable with a simple mixture of the constituent parts, and are therefore relevant in a wide range of applications [1,2]. Silicon-based sol-gel chemistry is recognized as one of the main routes for the synthesis of hybrid materials owing to the wide availability of molecular precursors and the stability of the Si–C bond [3]. Silsesquioxanes (SSQs) are compounds with the general formula (RSiO_1.5_)*_n_*, where R is an organic functional group or a H atom, and *n* is the number of repeating units that can be obtained from the hydrolysis-condensation of organotrialkoxysilanes (RSi(OR’)_3_) [4,5]. While SSQs are routinely synthesized following conventional hydrolytic sol-gel processes, a higher degree of control on the structure of the final material can be achieved with the nanobuilding blocks (NBBs) approach [6,7,8,9]. Indeed, the assembly of pre-formed nano-objects based on different SSQs architectures, such as cage and ladder-like structures (Supplementary Materials, Figure S1), can be used for producing the final material, allowing for a bottom-up design of its properties [4,5,10].

**Figure 1:**
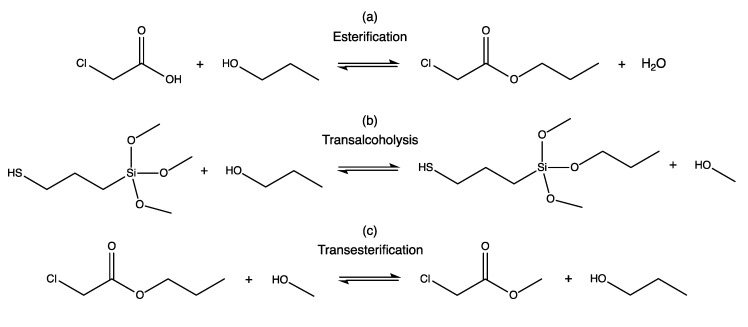
Reactions occurring in the ISWP reaction mixture (adapted with permission from [15] Borovin et al., 2016 © Wiley-VCH).

Some of us previously reported the sol-gel synthesis of silsesquioxane NBBs by the in situ water production (ISWP) route, where the water necessary for the hydrolysis of the silane precursor is provided by the esterification reaction between a carboxylic acid and an alcohol [11,12,13]. The extent of hydrolysis–condensation in dependence on the kinetics of water production was assessed by time-dependent monitoring of the reactions, mainly through infrared spectroscopy (FTIR), nuclear magnetic resonance (NMR), and gel permeation chromatography (GPC) analyses, demonstrating the ability to tune SSQs’ size and architecture by simply controlling the reaction duration. In particular, in the case of thiol-functionalized SSQs (SH-NBBs) [14], obtained by controlled hydrolysis-condensation of 3-mercaptopropyltrimethoxysilane (McPTMS) with chloroacetic acid (ClAAc) in 1-propanol solution at 100 °C (Figure 1a), the well-defined octakis (3-mercaptopropylsilsesquioxane) is the major species between 70 and 100 h reaction time; on the contrary, the ladder-like species appeared predominant for lower duration, according to ^29^Si NMR spectroscopy [15]. However, the ISWP route is characterized by the occurrence of different reactions (Figure 1b,c), leading also to side products such as chloroacetic acid-derived compounds that cannot be completely removed from the SH-NBBs solution.

Despite the confirmed solution stability under prolonged storage and the substantial keeping of thiol functions in the final SH-NBBs, the presence of by-products can be detrimental towards both assembly and further SSQs functionalization in the final hybrid O/I materials. Attempts to purify sol-gel-prepared SSQs were reported in the literature generally with unsatisfactory results [9]; in the case of SH-NBBs, the elimination of by-products by sol evaporation under reduced pressure was unsuccessful [14].

The reactive SH- function is appealing for tuning the surface functionality of O/I hybrids, and can be exploited for different applications, such as immobilization and the sensing of biomolecules and trace metals [16,17,18], or the development of coatings with different wettability [19]. SH-NBBs sols demonstrated good filming ability onto glass and Si substrates [15], and this evidence prompted us to investigate their use for modifying the surface features of cotton fabrics and paper, for their possible application in oil-water separation. Microporous polymers, such as PU foams and PDMS sponges, and carbon-based materials present excellent adsorption characteristics due to oleophilic and hydrophobic properties [20]. However, adsorbent materials with low environmental impact in terms of waste disposal and eventual recyclability are envisaged, such as natural materials with improved oleophilic and hydrophobic behavior.

In this paper, a two-step functionalization process of both cotton fabrics and paper has been studied. In a first step, SH-NBBs coatings were prepared on cotton and paper substrates; in the second step, an initiator-free click thiol-ene coupling reaction was exploited for coating functionalization with long alkyl chains in order to further increase the hydrophobic character of the exposed surface. Scanning Electron Microscopy and Confocal Microscopy were used for coating characterization and, depending on the specific nature of the substrates, surface wettability was evaluated by shedding angle and dynamic contact angle measurements.

Moreover, according to the NMR study, the purification of SH-NBBs was also achieved during the coating procedure, thus enabling the preparation of long alkyl-chain functionalized NBBs solutions via click chemistry. This approach allows to overcome the difficulty in obtaining functionalized SSQs oligomers directly starting from trialkoxysilanes with long alkyl chains, such as octyltriethoxysilane, since these precursors own very low hydrolysis-condensation reactivity [21,22].

## 2. Results and Discussion

### 2.1. SH-NBBs Coatings

SH-NBBs solutions prepared via ISWP at a different reaction time were used for coating cotton and paper substrates. On the basis of the NMR study performed for elucidating the SH-NBBs growth in solution [15], three reaction times were selected as representative of different SSQs architectures. After 6 h reaction the condensation degree is limited and SH-NBBs are represented by small linear and cyclic species mainly based on T^2^ units, i.e., according to the ^29^Si NMR notation, SiCO_3_ species with two bridging oxygen and one terminal OH function. These species after 16 h start to rearrange into fully condensed T^3^ units (with three bridging oxygen) mainly in ladder–type architectures. At 80 h SH-NBBs are mainly composed of condensed units with closed cages, i.e., octakis (3-mercaptopropylsilsesquioxane), as the dominant structure.

**Figure 2:**
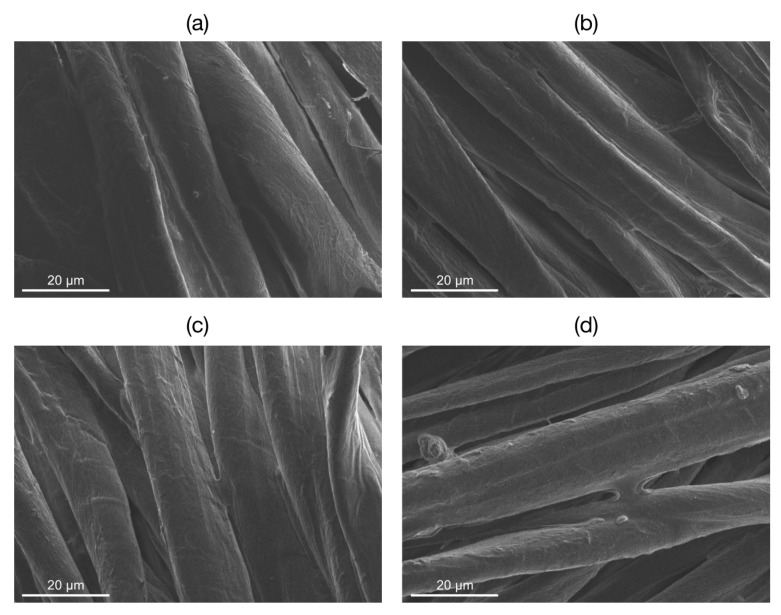
Scanning Electron Microscopy (SEM) images of raw cotton (**a**); *Cotton-NBB6h* (**b**); *Cotton-NBB16h* (**c**) and *Cotton-NBB80h* (**d**). Images were acquired at the same level of magnification.

The raw cotton and the cotton samples coated with SH-NBBs sols obtained after 6, 16, and 80 h reaction were characterized by Scanning Electron Microscopy, in order to assess the morphological changes undergone by the material surface during the first step of the process (Figure 2). In raw cotton, both the tubular and the secondary micro-fibrillar structures of the cotton fibers [23] can be observed. In *Cotton-NBB* samples the presence of a coating appears superimposed to the wrinkled surface textures and the appearance of sub-micrometric bumps, clumps, and bridge-like structures among the fibers can be clearly observed. From Figure 1, the filming ability of SH-NBBs solutions appear good independently of the duration of the ISWP reaction, thus assuring the effective coating of the cotton fibers in all samples.

To ascertain the presence and homogeneity of SH-NBBs coating on the fibers, EDX spectra were acquired on raw and coated cotton samples. In the case of SH-NBBs-coated samples, the signals due to Si and S, which are indicative of the presence of thiol-functionalized silsesquioxanes, can be clearly detected in the survey EDX spectra. On the contrary, the amount of Cl, which is indicative of chloroacetic acid and its esters i.e., the side products of the ISWP synthesis, appears very low and a clear correlation with the coating cannot be made on the basis of the acquired maps.

**Figure 3:**
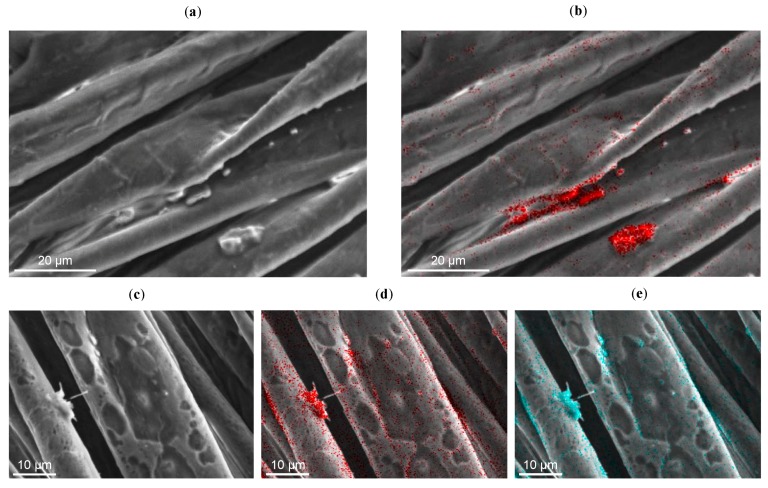
(**a**) SEM image of *Cotton-NBB80h*; (**b**) Si map of the corresponding area; (**c**) damaged *Cotton-NBB80h* specimen after prolonged SEM observation, and elemental maps for Si (**d**) and S (**e**) of the sample zone shown in (**c**).

In *Cotton-NBB16h,* from the element maps obtained analyzing different selected zones, the Si and S signals do not appear uniform but are stronger in conjunction with the bridge-like structures. The SEM-EDX analysis of *Cotton–NBB80h* (Figure 3a,b) points out that, in comparison with *Cotton-NBB16h*, S and Si are more visible in the areas of the fibers where bridge-like structures or lumps are not present. These results can be probably explained by taking into account the viscosity of the SH-NBBs solutions, which increases with an increasing reaction time as a result of oligomers growth.

This evidence is confirmed by the EDX results (that were recorded on a sample, exposed for a long time to the focused electron beam, and showed clear signs of damage compatible with coating degradation). Figure 3c,d and e suggest that the coating prior to degradation was more homogeneous than what was concluded on the basis of Figure 3a,b.

**Figure 4:**
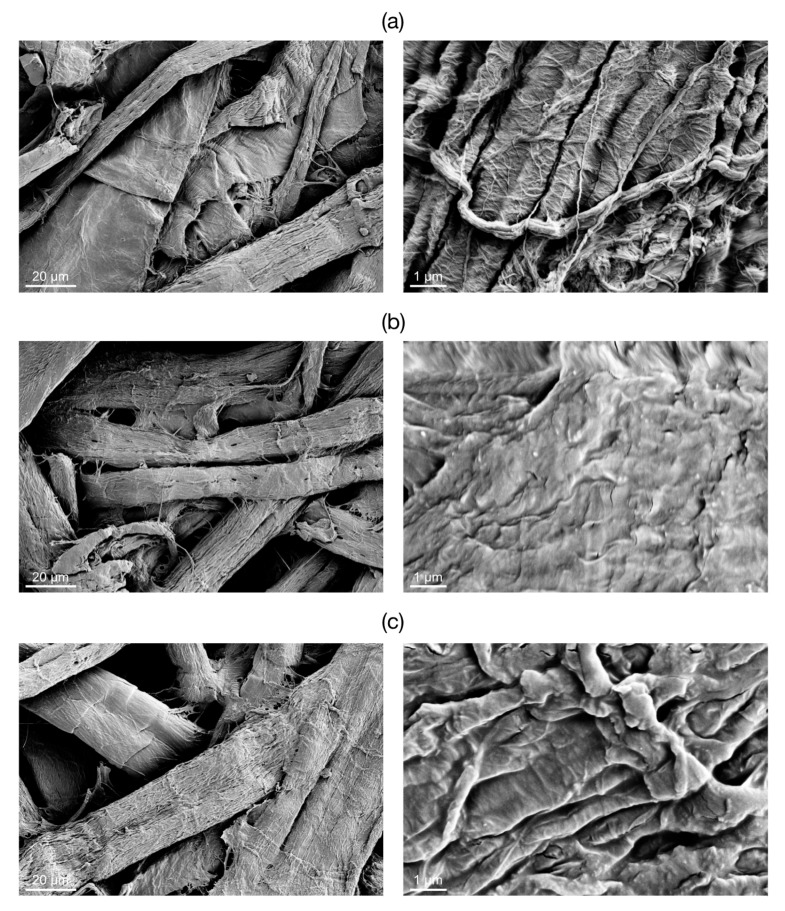
Low (left) and high (right) magnification SEM images of (**a**) the raw paper substrate and *Paper-NBB80h* samples, before (**b**) and after (**c**) rinsing in ultrasound bath. Images on the left were taken at the same level of magnification of those in Figure 1; images on the right were taken at a constant higher level of magnification.

The SEM images of the raw paper substrate and *Paper-NBB* samples are shown in Figure 4. Owing to the high heterogeneity of the substrate, it is difficult to clearly distinguish signs of the NBB coating from the low magnification images (Figure 4 left). However, the images recorded at higher magnification (Figure 4 right) show the SH-NBBs layer covering the disordered fibrillary surface texture of the raw paper substrate. This layer exhibits a sub-micrometric roughness that persists even after rinsing of the samples in the ultrasound bath (see Section 3.2).

From the SEM study, both *Cotton-NBB* and *Paper-NBB* samples show that coating with SH-NBBs leads to changes in surface morphology with the appearance, in the case of cotton fabrics, of bridge-like structures connecting the fibers. However, from EDX spectra the presence of SH-NBBs is detected also in the other regions, which suggests that a fairly homogeneous layer is present on the majority of the natural substrates. Therefore, a quite good distribution of the thiol anchoring sites for the subsequent alkene functionalization is also expected.

In the case of *Paper-NBB*, it is worth noting that the coating roughness is enhanced as a consequence of its rinsing using an ultrasound bath to remove the SH-NBBs excess. While the substrate fibers coating is still preserved, the change in morphology could be beneficial for the wetting behavior. Indeed, the sub-micrometric texture coupled with the microstructure given by the cellulosic fibers of cotton and paper might give rise to hierarchical multi-scale roughness, suitable for achieving extreme wetting properties. Finally, it should be mentioned that, despite the presence of the SH-NBBs coating, both cotton and paper samples keep their macroscopic features, such as color and mechanical behavior, and exhibit quite negligible increases in weight.

The very low amount of deposited SSQs makes the structural characterization of the SH-NBBs coating and the study of the interface between substrate and coating very difficult. However, some hints on the structural features displayed by the coating were obtained by solid state NMR experiments. In particular, a ^29^Si NMR spectrum was recorded on a cotton sample, prepared ad hoc by repeated immersion steps in the SH-NBBs solution reacted for 80 h (Supplementary Materials, Figure S2); 36 k scans were acquired in a cross-polarization experiment (CPMAS). The main signal, due to fully condensed T^3^ units with the second resonance related to T^2^ units, can be observed in the spectrum. The CPMAS sequence does not lead to quantitative results since it is well known that the magnetization transfer enhances the intensity of proton-rich environments. Accordingly, the low intensity of the T^2^ signal is an indication of the high crosslinking degree of the SSQs coating.

### 2.2. SH-NBBs Purification and Thiol-Ene Click Functionalization

As reported in the experimental section (Section 3.2), after the immersion of cotton and paper substrates in the SH-NBBs solution the coated samples were rinsed in chloroform in order to remove the ungrafted SH-NBBs, the solutions were kept to dryness, and the obtained solids were dissolved in deuterated chloroform (*SH-NBB_c* and *SH-NBB_p* solutions) for the NMR analyses.

The structural features of SH-NBBs and ISWP by-products were already described in Borovin et al. [15]. Accordingly, it has been proved that the synthesis of SH-NBBs leads to a final solution containing propyl- (PrClA) and methyl-chloroacetate (MeClA) residues, as a result of esterification and transalcoholysis reactions (Figure 1), and chlorothioacetate derivatives. The latter compounds are formed both through direct reaction between McPTMS derivatives and ClAAc, or by transesterification between McPTMS derivatives and propyl or methyl chloroacetate [15]. In order to remove the side-products from the pristine SH-NBBs solution, purification methods (as extraction or volatilization under reduced pressure) were proved to be not effective, representing an obstacle for further reactions. As a matter of fact, the attempt at running the thiol-ene coupling using the as prepared SH-NBBs solution was unsuccessful, contrary to the observed reactivity of the McPTMS precursor in 1-propanol solution.

**Figure 5:**
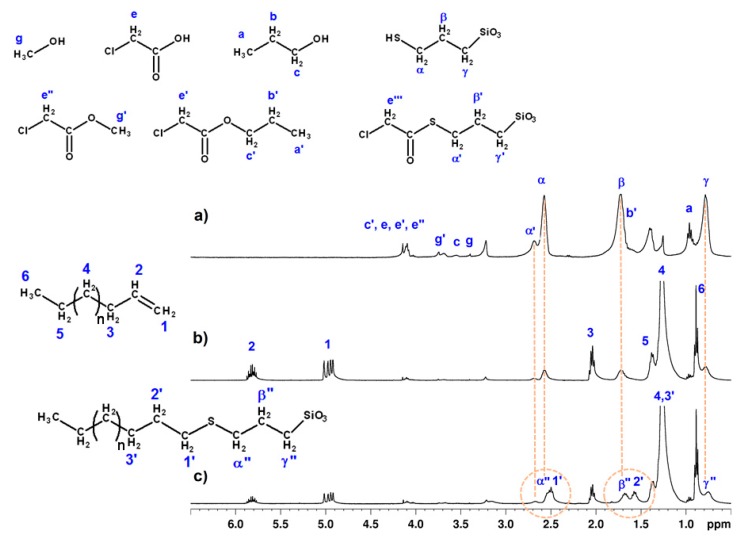
^1^H NMR spectra and peak assignment of (**a**) *SH-NBBs_c* solution obtained by rinsing *Cotton-NBB80h*; a mixture of *SH-NBBs_c* and C14 before (**b**); and after (**c**), 30 min irradiation at λ_max_ = 254 nm.

Therefore, the NMR spectroscopy was used not only to verify the structural integrity of SH-NBBs recovered by rinsing in chloroform the coated samples, but also to assess both the purification process and the effectiveness of thiol-ene functionalization of the purified SH-NBBs. Figure 5a shows the ^1^H NMR spectrum of the *SH-NBB_c* solution prepared by rinsing *Cotton-NBB80h*, as an example of the results obtained with the different *SH-NBB_c* and *SH-NBB_p* solutions. The peak assignment is based on the numbering scheme shown in Figure 5.

The most intense signals are attributed to α, β, and γ resonances of the mercaptopropyl chain, and together with α’, due to the chlorothioacetate derivative of the pristine propylsilane group [15], confirm the presence of SH-NBBs in the rinsing residue and, according to the chemical shifts, prove the maintenance of the structural features displayed by the pristine SSQs. Moreover, the integrated areas of (α + α’), β, and γ resonances are an indication that the mercaptopropyl functionalities of the recovered SH-NBBs do not undergo any degradation.

The resonances due to the ISWP side products are still observable in the spectrum. In detail, signals c, e, e’, and e” are due to chloroacetic acid and its esters; in addition, PrClA gives rise to a’ and b’ resonances, the latter appearing as a shoulder of the signal, while MeClA gives rise to g’ resonance. The low intensity of c and g signals suggest the large removal of 1-propanol and methanol during cotton fabrics processing. Finally, other signals not ascribable to SH-NBBs or to ISWP side-products can be observed and have been tentatively assigned to products of cotton degradation.

In order to evaluate the amount of residual ClAAc derived compounds with respect to the SH-NBBs, the following parameter can be calculated (Equation (1)):

(1) $$R_{\mathrm{CI}}=\frac{c^{\prime }+e+e^{\prime }+e^{\prime \prime }}{\alpha +\alpha^{\prime }}$$

where the different quantities are the integral areas of the specified signals. The parameter calculated according to Equation (1) slightly overestimates the molar ratio of ClAAc and its esters with respect to SH-NBBs, since the resonances due to SH-NBBs (α and α’), ClAAc (e), and MeClA (e”) each contribute two protons to the signals, while PrClA contributes four protons (c’ and e’). The calculated R_Cl_ values (Table 1) for *SH-NBB_c* and *SH-NBB_p* solutions indicate the effectiveness of the SH-NBBs purification following the adopted procedure. Indeed, taking into account the 6:1 molar ratio between ClAAc and McPTMS used for the SH-NBBs synthesis, the nominal R_Cl_ for the as-prepared solutions would assume values of 6 and 12, if only ClAAc and MeClA or PrClA were present, respectively. Conversely, in *SH-NBB_c* and *SH-NBB_p*, R_Cl_ assume values ranging from 0.061 to 0.165, resulting in a more than fifty-fold reduction in the relative amount of ClAAc and its esters.

In order to quantitatively evaluate the reliability of the purification step, the percentage of unreacted available thiol groups can be calculated according to Equation (2):

(2) $${\mathrm{SH}}_{\mathrm{av}}(\%)=\frac{\alpha }{\alpha +\alpha^{\prime }}$$

The SH_av_ values (Table 1) are generally close to 100% for both paper and cotton supports, with the exception of the purified SH-NBBs 80 h that present a loss of thiol functionalities higher than what was previously reported for the as-prepared SH-NBBs solutions [15]. However, this result can be related to the fact that the solutions were kept at room temperature during the coating procedure, thus allowing the thioesterification reaction to proceed.

From the above results, the SH-NBBs purification appears effective, and the process is independent of the coated substrate and highly reproducible. In conclusion, the employed NBB coating procedure of cotton and paper produces recyclable wastes, from which the purified SH-NBBs can be easily collected and further reacted.

The *SH-NBB_c* and *SH-NBB_p* solutions were mixed with 1-decene or 1-tetradecene to study the NBBs reactivity in the thiol-ene coupling run without any photoinitiator addition. Figure 5b,c shows the ^1^H NMR spectra of *SH-NBBs_c* (obtained from *Cotton-NBB80h*) and C14 mixture before and after irradiation for 30 min with λ_max_ = 254 nm. According to the signal labeling presented in Figure 5, in the mixture before irradiation C14 can be easily identified by the strong peaks at 1.27, 2.04, and 1.37 ppm due to the methylene protons 4, 3, and 5, respectively, in the alkyl chain, the methyl signal (6) at 0.88 ppm, and the two multiplets at 5.80 and 5.00 ppm due to the double bond end group (1 and 2). After UV irradiation, it is possible to observe a decrease in signal intensity of 1, 2, and 3 resonances and the corresponding appearance of signals 1’ at 2.50, 2’ at 1.57, and of resonance 3’ at 1.26 (overlapped with 4), which is proof of the conversion of C14 into the thioether product of thiol-ene coupling. Moreover, the formation of the thioether group leads to shielding of the α, β, and γ nuclei, with a consequent up-field shift of 0.03 ppm of the mercaptoproyl signals (α”, β”, and γ”). The reaction gives rise to methylene groups with similar neighboring, thus leading resonances 1’ and 2’ to appear overlapped to α” and β”, respectively.

Since deconvolution of α” and 1’ signals with Gaussian-Lorentzian line-shapes failed to obtain an acceptable fit, and integral areas of β” and 2’ are not reliable due to the presence of other signals in the same range (the yield of the reaction was calculated from the comparison of the spectra before and after irradiation). The intensities of α, α”, and 1’ resonances were normalized in the spectra with respect to the signal 6 at 0.88 ppm, which is unaffected by the reaction. The integrated areas are used in Equation (3) that gives the thiol-ene click reaction yield in %:

(3) $${\mathrm{Yield}}_{\%}=100\times [{\left(\alpha +\alpha^{\prime }+1^{\prime }\right)}_{\mathrm{after}}-\alpha_{\mathrm{before}}]$$

Upon irradiation at λ_max_ = 254 nm in the presence of C14, an almost complete conversion is detected for *SH-NBB_c* and *SH-NBB_p* at 6, 16, and 80 h, which gives a yield above 99% with the only exception of *SH-NBB_p* at 16 h that reaches 93%. The same results are obtained for the reaction with 1-decene, indicating that the conversion reaction is achieved independently from the alkyl chain length. On the contrary, from the ^1^H NMR spectra no reaction is observed with the same samples irradiated at 365 nm or kept in the darkness (control samples). All the obtained results are summarized in Table S1.

The ^1^H NMR study of purified SH-NBBs/alkene mixtures demonstrates that initiator-free thiol-ene coupling between SH-NBBs and selected alkenes in over-stoichiometric amounts occurs with near to quantitative conversion after irradiation with light in the middle UV range with λ_max_ = 254 nm. Moreover, in accordance with its click character, the observed thiol-ene reaction is very fast, achieving almost complete completion within 15 min, as demonstrated for *SH-NBBs_p*/C14 mixtures. To the best of our knowledge, this constitutes the fastest and most efficient example of initiator-free thiol-ene coupling of silsesquioxane-based materials ever achieved [24,25].

While reactions conducted under irradiation at λ_max_ = 254 nm achieved excellent yields typical of click reactions, samples irradiated with λ_max_ = 365 nm did not react at all for the given exposure times (although, from this result it cannot be concluded that thiol-ene coupling cannot occur in those conditions; the reaction would certainly proceed much more slowly than for a λ_max_ = 254 nm irradiation, in accordance with the literature [26,27]).

### 2.3. Thiol-Ene Functionalization of SH-NBBs Coated Cotton and Paper

In the second functionalization step, *Cotton-NBB* and *Paper-NBB* samples were soaked in the pure alkene without the addition of any photoinitiator, and subsequently irradiated with λ_max_ = 254 nm. As reported above, the structural characterization of the coating by solid state NMR was possible only using the sample ad hoc prepared. With the purpose to check the occurrence of the click coupling between the alkene and the available thiol functions of the coatings, ^1^H MAS NMR spectra were recorded on the previous sample before and after the reaction. Comparing the spectra of raw and coated cotton (Figure S3a,b), the overlapping of the relatively sharp peaks belonging to the NBBs and the broad cellulose signal can be appreciated. Moreover, the signal due to the substrate further broadens as a consequence of the interaction with the NBBs. The spectrum recorded on the coated sample soaked in C14 after UV irradiation (Figure S3c) presents two sharp and intense peaks at 1.3 and 0.9 ppm, which prove the presence of the long alkyl chain; the absence of sharp resonances in the region 5–6 ppm, attributable to the double bond protons (Figure S3c), confirms the occurrence of the click reaction.

In order to observe the effect of the click functionalization step on the coating, the SEM analysis was run on *Cotton-NBB click* (Figure S4) and *Paper-NBB click* (Figure S5) samples. *Cotton-NBB click* samples appear to have lost the bridge-like connections between fibers observed in their parent coated samples, but at higher magnification the sub-micrometric features previously observed on the coatings (Figure 2) appear clearly visible. Similar results are obtained for *Paper-NBB click* samples. Therefore, even if some large-scale features are lost with the click functionalization, the coating integrity is generally assessed on the basis of the SEM analysis.

Aiming to estimate the spatial distribution of available thiol groups and, consequently, the extent of alkene functionalization, the confocal microscopy analysis of *Cotton-NBB80h*, *Cotton-NBB80h C14 click* and raw cotton reference samples was conducted after binding with 7-diethylamino-3-(4-maleimidophenyl)-4-methylcoumarin fluorophore (CPM). An important characteristic of CPM is that it becomes fully fluorescent in the blue spectral range only when bound to thiols, while it is only weakly fluorescent in the absence of binding.

**Figure 6:**
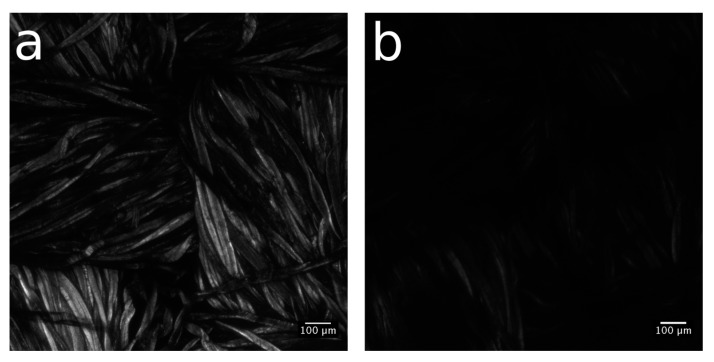
Confocal microscopy image of (**a**) *Cotton-NBB80h* and (**b**) *Cotton-NBB80h C14 click* samples after CPM treatment.

The raw cotton sample (shown in Figure S6a) appears brighter and with a more homogeneous emission than the *Cotton-NBB80h* sample (Figure 6a), while practically no fluorescence is visible in *Cotton-NBB80h C14 click* (Figure 6b). The brightest emission of raw cotton is produced by the cotton autofluorescence, which lies in the same spectral region of CPM emission. As it is common for natural fibers, cotton fluorescence has its origin in residual nucleotides, such as nicotinamide adenine dinucleotide phosphate (NADPH), and in the structure of cellulose, though the molecular origin of the latter phenomenon is still poorly understood [28,29]. Therefore, while it is not possible to determine the extent of CPM binding to SH-NBBs, the lower emission of *Cotton-NBB80h* can be reasonably ascribed to the quenching of cotton self-fluorescence resulting from the surface functionalization with SH-NBBs. Moreover, the evident reduced emission after click coupling (Figure 6b) can be taken as proof of the presence of a further functionalization of the surface, which derives from the reaction of the thiol groups available on the coating with the alkene.

**Figure 7:**
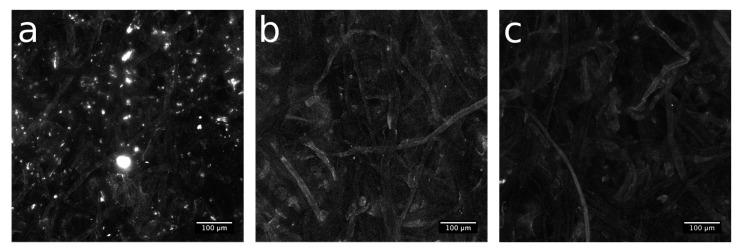
Confocal microscopy image of (**a**) *Paper-NBB80h*, (**b**) *Paper-NBB80h click 15’,* and (**c**) *Paper-NBB80h click 30’* samples after CPM treatment.

Even if raw paper exhibits a strong autofluorescence signal (Figure S6b), the spatial distribution of available thiol groups in the NBB coating before and after the click coupling was evaluated by means of confocal microscopy, after reaction with fluorescein diacetate 5-maleimide (FDM). As visible in Figure 7a, *Paper-NBB* exhibits diffused fiber fluorescence, with high intensity bright spots that could be addressed to a high local confinement of the FDM dye. After the click reaction (15’ and 30’, Figure 7b,c, respectively) the bright spots are switched off. Moreover, the overall fluorescence emission evidences a tendency to reduce with the UV exposure time and, thus, with the progression of the click reaction that reduces the available SH groups at the surface.

Owing to the different characteristics of the substrates, the performance of the two-step surface functionalization as a hydrophobization treatment was evaluated with different techniques for cotton and paper substrates.

**Figure 8:**
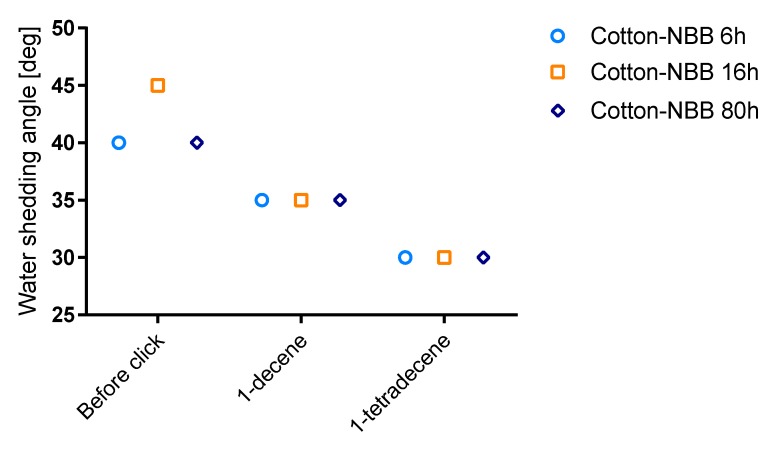
Results of the water shedding angle measurements of *Cotton-NBB* samples before and after click functionalization with either C10 or C14.

Wettability of cotton samples was evaluated by the means of water shedding angle (ω) measurements, a technique specifically developed for rough, non-reflective textile surfaces such as cotton [30]. The results of the analysis are summarized in Figure 8; raw cotton samples showed ω values higher than 70° and are not reported in the figure. ω decreases both after NBB coating and click functionalization of the samples. Only a small difference was observed among coated samples, with *Cotton-NBB6h* and *Cotton-NBB80h* showing better performance than *Cotton-NBB16h*, but the difference disappeared after click functionalization. It is worth of noting that C14 functionalized samples appear to be more hydrophobic than those functionalized with C10.

However, when water droplets are pinned on *Cotton-NBB* samples, after a very short time they are absorbed into the fabric. On the other hand, droplets pinned on the click functionalized samples remain indefinitely on the fabric surface. The behavior of *Cotton-NBB* samples that show an instaneous hydrophobic character suggests that the coating, although characterized by sub-micrometric surface roughness, is not sufficient to permit a stable surface hydrophobicity. On the other hand, after click functionalization, the introduced long alkyl chains, together with the hierarchical roughness conferred by the coating, can satisfy both the chemical and morphological conditions for stable hydrophobicity of the surface. Indeed, at least for the samples reacted with C14, the obtained ω values are comparable with those reported in the literature for superhydrophobic cotton fabrics obtained by silicon nanofilament coating [31].

The higher hydrophobicity of C14 functionalized samples could be related to the interactions occurring among the alkyl chains, which can increase with the functionalization density [32]. The grafted alkyl chains are free to rotate and can fold to find the best accommodation over the material surface, thus offering a degree of hydrophobic coverage that is probably higher for C14 than for C10.

**Figure 9:**
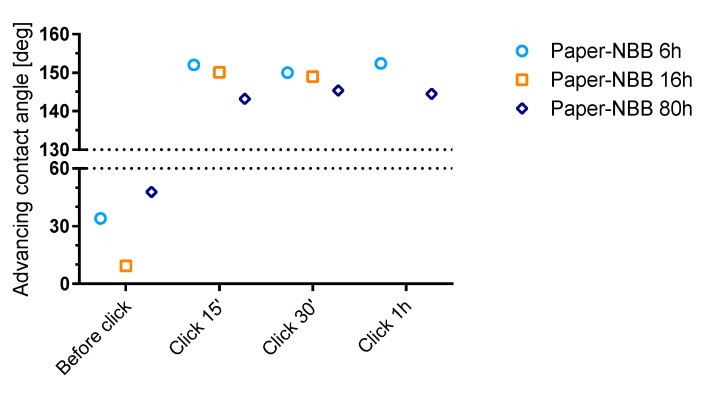
Results of the advancing contact angle of *Paper-NBB* samples before and after click functionalization with C14 with increasing irradiation time. Error bars are smaller than the used data symbols.

Water contact angle on paper samples was evaluated by the means of the Wilhemly technique. Force curves were analyzed to evaluate the advancing contact angle (Figure 9). The receding angle (ϑ_rec_) was impossible to evaluate because samples resulted completely wet after the advancing phases. This fact is explained by water adsorption due to defects on the sample edges and the capillary rise into the paper fibers network.

Raw paper samples, as expected, appeared to be completely hydrophilic with ϑ_adv_ compatible with 0° (not reported in the Figure 9). ϑ_adv_, which slightly increased after NBB coating, was found to significantly increase after click functionalization, reaching values greater than 140°.

The high values of ϑ_adv_ observed in *Paper-NBB click* samples, typical of highly hydrophobic material, confirm the effectiveness of the proposed two-step surface functionalization process as a hydrophobization procedure. As observed for cotton samples, it appears that no substantial difference exists between samples coated with SH-NBBs prepared at different reaction times, although *Paper-NBB6h click* shows the highest ϑ_adv_ values. Moreover, it can be clearly seen that no remarkable change is observable with increasing irradiation times, and 15 min is a sufficient time to achieve the maximum observed hydrophobicity. This result would be in good accordance with the fast kinetics of the thiol-ene coupling between SH-NBBs and the C14 observed in solution.

According to the results obtained on cotton and paper, the click functionalization step is effective in introducing hydrophobic alkyl chains on the material surface while keeping the surface roughness obtained as a result of the NBB coating step. Consequently, at the end of the two-step surface functionalization, samples possess both the chemical and morphological requirements for extreme wettability. Therefore, the two-step surface functionalization process here proposed is a viable process for obtaining the efficient hydrophobization of natural materials by combined sol-gel and initiator free thiol-ene chemistry. In perspective, the preparation of superhydrophobic materials without employing fluorinated compounds and based on cellulose appears as a good response to the request of low environmental impact materials for water remediation.

## 3. Materials and Methods

### 3.1. Materials

Chloroacetic acid (ClAAc, pure), alkenes: 1-decence (C10, >97%), 1-tetradecene (C14, >97%); chloroform (purum, >99.5%), chloroform-d (>99.8 atom %D, with 0.05% (*v*/*v*) tetramethylsilane), dimethyl sulfoxide (DMSO, >99.5%), 7-diethylamino-3-(4-maleimidophenyl)-4-methylcoumarin (CPM, >90%), and fluorescein diacetate 5-maleimide (FDM, for fluorescence) were acquired from Sigma-Aldrich (St. Louis, MO, USA); 3-mercaptopropyltrimethoxysilane (McPTMS, >95%) and dibutyltin dilaurate (DBTL) were acquired from ABCR (Karlsruhe, Germany); 1-propanol (PrOH, Baker analysed >99%) was acquired from Fischer Scientific (Hampton, NH, USA). White, commercially available plain-weave cotton fabrics were used without any further treatment; 70 mm diameter Whatman Schleicher & Schuell ashless 589/2 white ribbon filter paper (Maidstone, UK) was used as the paper substrate without any further treatment.

### 3.2. SH-NBB Coatings and Purification

Thiol-functionalized silsesquioxane NBBs (SH-NBBs) were synthesized according to previous works [14,15]. ClAAc (1275.8 mg) was first brought to 100 °C and melted in an open borosilicate glass tube under N2 fluxing. PrOH (3.027 mL) was then added as a solvent, followed by the trialkoxysilane precursor McPTMS, (418 μL) and finally by DBTL (20 μL) as a condensation promoter. The tube was then sealed, obtaining a self-condensing reaction vial, and the mixture was left to react for a given time (6 h, 16 h or 80 h). The obtained clear, faintly yellow solutions (SH-NBB solutions) were stored at 4 °C in order to prevent any further reaction.

Raw cotton fabrics were immersed for 24 h into the SH-NBB solutions and subsequently left to dry overnight in air at room temperature on a horizontal stainless steel drying rack. The fabrics were then rinsed in chloroform for 1 h in order to remove the ungrafted SH-NBBs. The obtained samples were labeled *Cotton-NBB.* NBB coating of paper samples was conducted following the same procedure as *Cotton-NBB* samples, with an additional rinsing with chloroform in an ultrasound bath for 7 min. The obtained samples were labeled *Paper-NBB*.

The chloroform solutions obtained by rinsing both *Cotton-NBB and* *Paper-NBB* samples were left to evaporate at room temperature under a fume hood for several days. The whitish precipitate was dissolved in chloroform-d, containing 0.05% (*v*/*v*) tetramethylsilane (TMS) as an internal standard for the ^1^H NMR characterization. The solutions obtained from *Cotton-NBB and* *Paper-NBB* samples were labeled *SH-NBBs_c* and *SH-NBBs_p,* respectively.

### 3.3. Thiol-Ene Click Functionalization

400 μL of *SH-NBB_c* or *SH-NBB_p* solutions were transferred in a 5 mm NMR tube and an appropriate alkene (C14 or C10) volume was added so that [SH-NBBs]:[alkene] = 1:1.75. Tubes were sealed and both irradiated (at λ_max_ = 254 nm or λ_max_ = 365 nm, 4 W, 2 cm distance from lamp) for 15 or 30 min, or kept in the darkness for 2 days (control). ^1^H NMR spectra were acquired before and after each procedure.

Coated cotton fabrics and paper were soaked in the pure alkene (C14 or C10) for 5 min, without any photoinitiator, and immediately transferred in a black box where they were irradiated continuously with λ_max_ = 254 nm for 1 h tilting the exposed side every 15 min. The samples were then rinsed in chloroform and left to dry in air at room temperature. Cotton samples were labelled *Cotton-NBB* C10 *click* or *Cotton-NBB* C14 *click*, respectively. Coated paper samples, soaked in C14 and irradiated for 15 and 30 min, and 1 h, were labelled *Paper-NBB click 15’*, *Paper-NBB click 30’, and Paper-NBB 1h.*

### 3.4. Characterization Techniques

Raw cotton and *Cotton-NBB* carbon coated samples were analysed with an Hitachi S-3400N microscope (Hitachi Ltd., Tokyo, Japan) with an accelerating voltage of 3 kV; EDX analysis were conducted with the built-in Oxford Instruments EDX instrument with an accelerating voltage of 10 kV. *Cotton-NBB click* samples were analysed after Pt/Pd sputtering with a Jeol JSM-5510 microscope (Jeol Ltd., Tokyo, Japan). Images at low magnification were taken with an accelerating voltage of 3 kV; images at a high magnification were taken with an accelerating voltage of 10 kV. *Paper-NBB* samples were analysed after Pt/Pd sputtering with a Supra 40 microscope (Carl Zeiss AG, Oberkochen, Germany). Images were taken with an accelerating voltage of 2 or 3 kV.

Solution-state NMR spectra were collected on a Bruker 400 WB spectrometer, equipped with a BBO liquid probe, or a Bruker 300 AvanceIII spectrometer, equipped with a 5 mm BBFO probe (Bruker Corporation, Billerica, MA, USA). The carrier frequencies were 400.13 and 300.13 MHz, respectively. ^1^H single pulse sequence with pi/6 flip angle was used (400: pulse = 10 µs/recycle delay = 5 s/32 scans; 300: pulse = 4 µs/recycle delay = 8 s/32 scans). The Bruker Topspin software was used for spectra analysis.

Confocal microscopy analyses were run on cotton samples treated with CPM florophore, whereas paper samples were reacted with FDM. 10 mg of CPM was dissolved in 1.6 mL of chloroform, obtaining a bright orange solution. Cotton fabrics were immersed in 500 μL of the obtained solution and samples were left to react for 2 h at 4 °C in the darkness. The bright orange cotton samples were removed from the solution and dried at ambient conditions in the darkness. Paper samples were stained with FDM [33]. 3 mg of FDM were first dissolved in 25 mL of DMSO and 275 μL of sodium phosphate buffer solution (pH = 7) were added. Paper samples were immersed in the obtained solution (5 mL) and left to react for 21 h at room temperature away from any light source. The samples were rinsed in chloroform for 30 min to remove excess solvent. Analysis on CPM treated samples was carried out with a Nikon A1 laser confocal microscope (Nikon Corporation, Tokyo, Japan), exciting the samples at 405 nm wavelength and collecting fluorescence emission at 450/50 nm. Conversely, confocal microscopy on FDM-treated samples was obtained exciting samples with λ = 488 nm laser source and collecting emission at 525/50 nm.

Multi-stack pictures were collected for each sample and then were post elaborated using Fiji image processing software [34]. Multi layers where Z-projected to get single maximum intensity images for each sample. Picture brightness was finally normalized to compensate for different pinhole apertures and photomultiplier gain settings, and to allow image comparison.

Water shedding angle measurements on cotton samples were conducted following the procedure described in the literature [30] on an apparatus built using a Standa 8MR151 motorized rotation stage (Standa Ltd., Vilnius, Lithuania) with automatic droplet dispensation constituted by a Harvard Instruments Nanomite MA1 70-2217 syringe pump (Harvard Apparatus Inc., Holliston, MA, USA) and a 100 μL volume Hamilton syringe (Hamilton Company, Switzerland). The following parameters were used: droplet volume equal to 10.0 ± 0.1 μL, needle-to-substrate distance set to 10.0 ± 0.5 mm, and minimum droplet path on sample equal to 20.0 ± 0.5 mm. Fifteen measurements were conducted for each inclination angle, with care taken that the impact of the drops on the samples occurred in a different place. If all the droplets rolled off the sample surface, inclination was decreased by 5°; the smallest angle for which all droplets rolled off the sample surface was addressed as the shedding angle.

Contact angle measurements on paper samples were conducted using a Dynamic contact angle analyzer (*Cahn DCA*-*322*) on rectangular specimens (10 × 25 mm) obtained by cutting the samples, setting an advancing/receding speed of 40 μm/s.

## 4. Conclusions

The in situ water production route provides the opportunity to control the size and architecture of silsesquioxanes, obtained through sol-gel reactions of trialkoxysilanes bearing reactive organic groups like the thiol function in the case of SH-NBBs [15]. Owing to the presence of side-products that cannot be efficiently removed from the solutions [14], further SSQs functionalization can present difficulties that limit the versatility of the synthesis route. The filming ability of SH-NBBs solutions obtained by ISWP at different reaction times has been exploited for coating both cotton fabrics and paper. A good degree of coating was generally achieved and the fairly homogenous deposited layers imparted to the natural materials a hierarchical roughness, necessary but not sufficient to extreme hydrophobicity. The ^1^H NMR analysis of the rinsing residues obtained during the SH-NBBs coating of both cotton and paper pointed out that the treatment yields recyclable wastes, which are constituted of purified SSQs. Therefore, the rinsing of coatings was proved to be a viable route to remove side-products as ClAAc and its derivatives, and gave the opportunity for the preparation of long alkyl chains substituted SSQs by means of fast, initiator-free thiol-ene click reactions with a nearly quantitative yield. To the best of our knowledge, this constitutes the fastest and most efficient example of initiator-free thiol-ene coupling of silsesquioxane-based materials ever achieved [24,25]. The functionalization of coated cotton and paper by thiol-ene click coupling was obtained via a simple immersion and UV-irradiation procedure, preserving the coating structure. The combination of multi-scale roughness, ensured by the sol-gel coating, and the hydrophobic surface character, imparted by the C10 and C14 chains, led the natural substrates to display stable highly hydrophobic behavior.

## Figures and Tables

**Table 1:** Summary of the values of R_Cl_ and SH_av_ (%), as calculated from the ^1^H NMR spectra of *SH-NBB_c* and *SH-NBB_p* solutions.

SH-NBBs	6 h		16 h		80 h	
Substrate	Cotton	Paper	Cotton	Paper	Cotton	Paper
R_Cl_	0.108	0.042	0.165	0.061	0.145	0.134
SH_av_ (%)	96.4	100	95.6	92.3	78.6	78.1

## Supplementary Materials

The following are available online at www.mdpi.com/1996-1944/10/8/913/s1, Table S1. Reaction yield for each purified SH-NBB/alkene mixture and irradiation procedure; Figure S1. Cross-linking morphologies of general silsesquioxane networks (reprinted with permission from V. Tagliazucca, E. Callone, S. Dirè, “Influence of synthesis conditions on the cross-link architecture of silsesquioxanes prepared by in situ water production route” J Sol-Gel Sci Technol (2011) 60:236–245, © Springer); Figure S2. ^29^Si CPMAS NMR spectrum of *Cotton-NBB80h* sample; Figure S3. ^1^H MAS NMR spectra of (a) raw cotton, (b) *Cotton-NBB80h* and (c) the product of click reaction with 1-tetradecene (*Cotton-NBB80h click*); Figure S4. SEM images of *Cotton-NBB6h C14 click* (a), *Cotton-NBB16h C14 click* (b), and (c) *Cotton-NBB80h C14 click*; Figure S5. SEM images of *Paper-NBB80h C14 click* samples after 15’ (a), 30’ (b) and 1h (c) exposure to UV radiation; Figure S6. Confocal microscopy pictures of autofluorescence emission from raw cotton (a) and raw paper (b).
