# Supplementary material for: Hydrophobic Coatings by Thiol-Ene Click Functionalization of Silsesquioxanes with Tunable Architecture
Source: Materials (Basel). 2017 Aug 8;10(8):913. doi: 10.3390/ma10080913 (PMC5578279; doi:10.3390/ma10080913)
Supplement: Supplementary file 1 [file materials-10-00913-s001.pdf]

Supplementary Materials: Hydrophobic Coatings by Thiol-Ene Click Functionalization of Silsesquioxanes with Tunable Architecture

Sandra Dirè, Davide Bottone, Emanuela Callone, Devid Maniglio, Isabelle Génois and François Ribot

[1] Department of Industrial Engineering, University of Trento, via Sommarive 9, 30123 Trento, Italy; sandra.dire@unitn.it; davide.bottone-1@studenti.unitn.it, emanuela.callone@unitn.it, devid.maniglio@unitn.it
[2] Sorbonne Universités, UPMC Univ. Paris 06 - CNRS - College de France, UMR 7574, Laboratoire de Chimie de la Matière Condensée de Paris, 4 place Jussieu, 75005 Paris, France; isabelle.genois@upmc.fr; francois.ribot@upmc.fr;
* Correspondence: Sandra Dirè, sandra.dire@unitn.it; Tel.: +39-0461-282456; Davide Bottone, current address: Department of Chemistry, University of Zurich, Winterthurerstrasse 190, 8057 Zurich, Switzerland; davide.bottone@uzh.ch, Tel: +41-44-63-54422

Silsesquioxane architecture

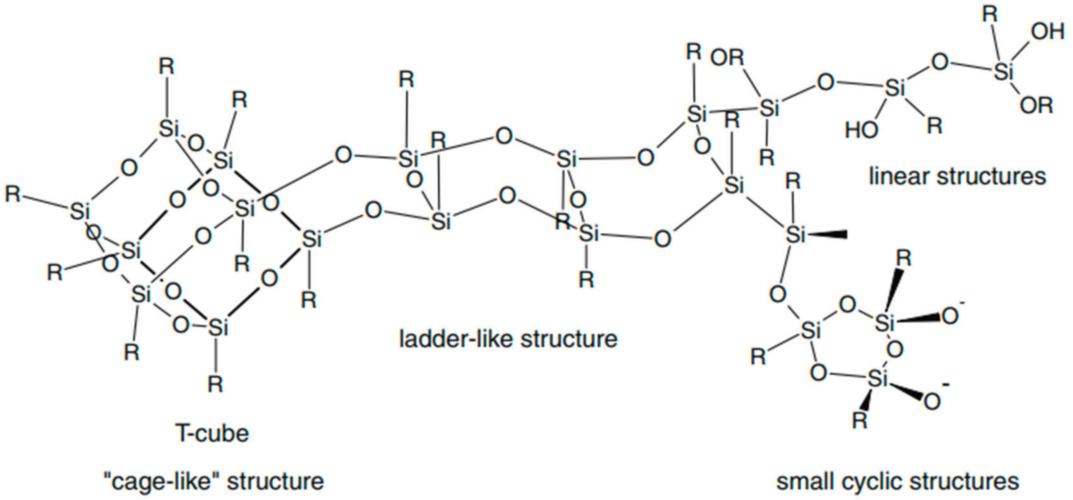

Figure S1. Cross-linking morphologies of general silsesquioxane networks (reprinted with permission from V. Tagliazucca , E. Callone, S. Dirè, "Influence of synthesis conditions on the cross-link architecture of silsesquioxanes prepared by in situ water production route" J Sol-Gel Sci Technol (2011) 60:236–245, © Springer)

Thiol-ene click reactions between purified SH-NBBs and long chain alkenes

The yield is calculated according to equation (3) from the ^{1}H NMR spectra shown in Figure 5.

Table S1. Reaction yield (%) for each purified SH-NBB/alkene mixture and irradiation procedure.

	SH-NBBs 6h		SH-NBBs 16h		SH-NBBs 80h	
	Cotton	Paper	Cotton	Paper	Cotton	Paper

254nm	100	93	100	99	99	100
365nm	-	-	-	-	Unreacted	-
Control	Unreacted	-	Unreacted	-	Unreacted	-

Solid State NMR analysis of *Cotton-NBB80h* and *Cotton-NBB80h click* samples

Experimental

Solid state NMR analyses were carried out with a Bruker 300WB spectrometer operating at a proton frequency of 300.13 MHz. NMR spectra were acquired with cp and sp pulse sequences under the following conditions: ^{29}Si frequency: 59.62 MHz, π/2 pulse 4.5 μs, contact time 5 ms; decoupling length 5.9 μs, recycle delay: 10 s, 36000 scans; ^{1}H frequency: 300.13 MHz, π/2 pulse 5 μs, recycle delay: 10 s, 8 scans. Samples were packed in 4 mm zirconia rotors, which were spun at 7 kHz under air flow. Q_8M_8 and water were used as external secondary references.

Results

The amount of coating on the cellulosic substrate is too low to be analysed at the solid state with NMR spectroscopy. Thus, in order to assess the coating features on cotton, a cotton sample was prepared ad hoc by repeated immersion steps in the SH-NBBs solution reacted for 80h and analysed through ^{29}Si CPMAS and ^{1}H MAS NMR.

Figure S2 shows the silicon spectrum of *Cotton-NBB80h* sample that is characterized by fully condensed $RSi(OSi)_3$ (T^3) and RSi(OSi)OH (T^2) units, at -65 and -56 ppm, respectively (R represents the mercaptopropyl chain). Due to the semi-quantitativeness of CPMAS experiment the intensity of T^2 units are strongly overestimated indicating that the condensation degree of the NBB is high as expected. The very low amount of Si in the whole materials does not permit a classical quantitative experiment and cause the low signal-to-noise ratio of the presented spectrum, besides the very high number of scans (36000).

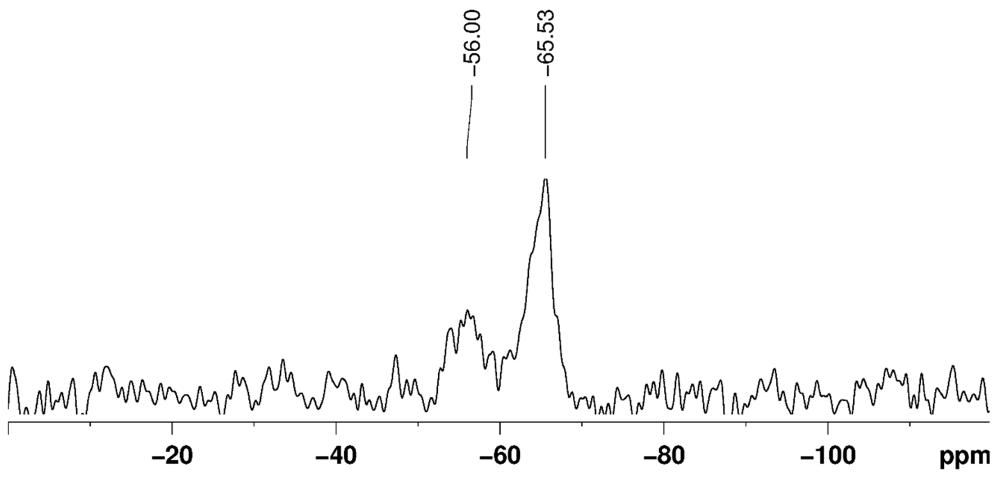

Figure S2. ^{29}Si CPMAS NMR spectrum of *Cotton-NBB80h* sample

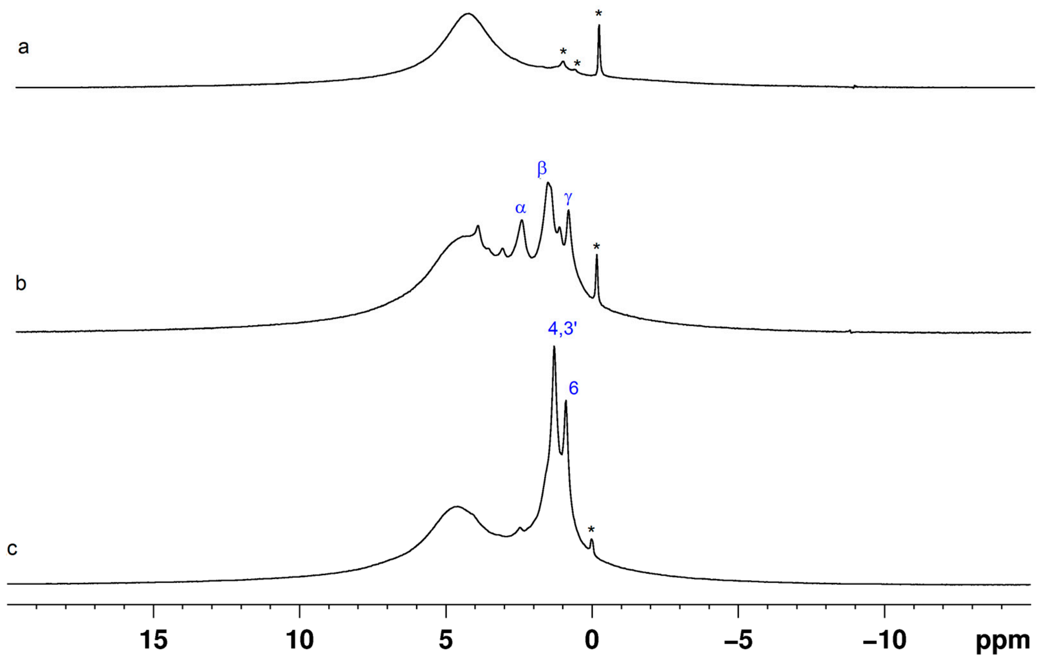

Figure S3. ^{1}H MAS NMR spectra of a) raw cotton, b) *Cotton-NBB80h* and c) the product of click reaction with 1-tetradecene (*Cotton-NBB80h click*). The peak marked with * are spurious.

The ^{1}H MAS NMR confirms the effectiveness of the coating. From the comparison between raw cotton (Figure S3a) and *Cotton-NBB80h* (Figure S3b) it can be appreciated the overlapping of the relatively sharp peaks in the 4-1 ppm range belonging to the pristine SH-NBBs, and the broad cellulose signal centered at 4.6 ppm, which experiences a further broadening due to the interaction with the NBBs. By exposing to UV radiation *Cotton-NBB80h* soaked in C14, the spectrum of sample *Cotton-NBB80h C14 click* (Figure S3c) presents two sharp and intense peaks at 1.3 and 0.9 ppm, which endorse the presence of the long alkyl chain. Moreover, the absence of sharp resonances in the region 5-6 ppm attributable to the double bond protons (Figure S3c) confirms the occurrence of the click reaction.

SEM images of *Cotton-NBB click* and *Paper-NBB click* samples

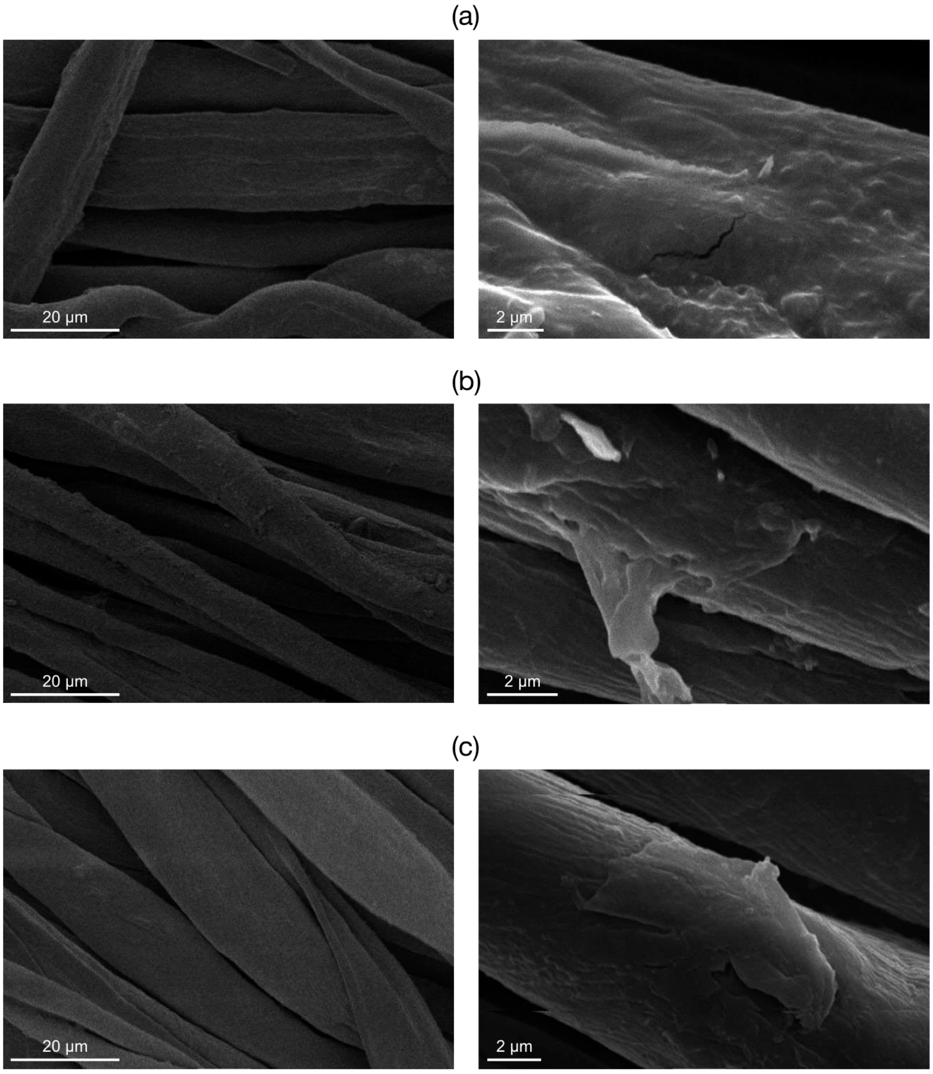

Figure S4. SEM images of *Cotton-NBB6h C14 click* (a), *Cotton-NBB16h C14 click* (b), and (c) *Cotton-NBB80h C14 click*.

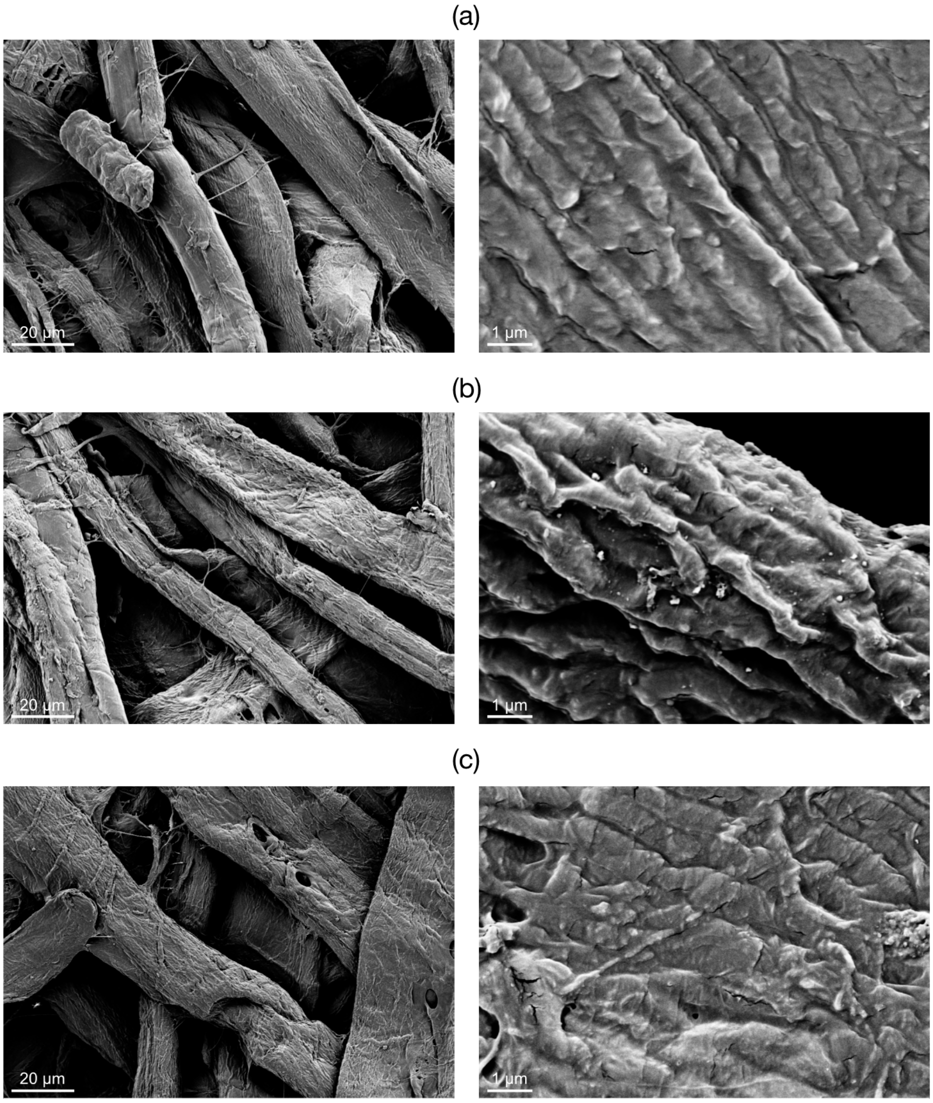

Figure S5. SEM images of *Paper-NBB80h C14 click* samples after 15′ (a), 30′ (b) and 1h (c) exposure to UV radiation.

Characterization of raw cotton and paper substrates by confocal microscopy

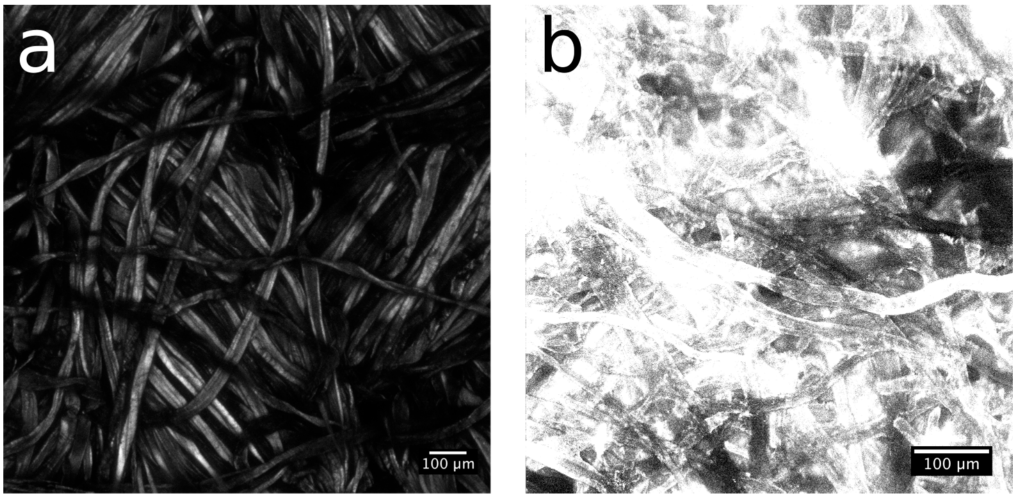

Figure S6. Confocal microscopy pictures of autofluorescence emission from raw cotton (**a**) and raw paper (**b**).
